# Exploring the capability approach to quality of life in disadvantaged population groups

**DOI:** 10.1038/s41598-022-18877-3

**Published:** 2022-09-15

**Authors:** Tomi Mäki-Opas, Richard Pieper, Marja Vaarama

**Affiliations:** 1 Department of Social Sciences, University of Eastern Finland (UEF), Yliopistonranta 1 E, Snellmania, P.O. Box 1627, 70211 Kuopio, Finland; 2 Gerontology Research Center GEREC, University of Tampere (TUNI), Tampere, Finland; 3 Department of Health and Social Management, UEF, Kuopio, Finland

**Keywords:** Human behaviour, Risk factors, Health policy

## Abstract

The capability approach argues that having effective choices and fair opportunities are essential to maintain and promote one’s own health and quality of life (QoL). This study examines the determinants of QoL in four disadvantaged population groups (young people not in education, employment, or training; long-term unemployed; refugees; older people living alone) within the framework of the capabilities by tracking the direct and indirect effects of individual and structural factors and capabilities on their QoL. Cross-sectional data (N = 866) with validated scales of quality of life (physical, social, psychological, and environmental QoL) and self-reported capabilities were utilized. Individual factors included age and gender and structural factors education and income. Descriptive statistics and structural equation modelling with latent variables were used for statistical analyses. Our results suggest that capabilities have crucial direct and mediating roles in achieving good QoL in the disadvantaged population groups. Individual factors had only small effects whereas especially the structural factors affected QoL through capabilities. Our results suggest that to reduce health inequalities and to promote wellbeing, policies should focus on improving both the structural factors and the individual capabilities of people in disadvantaged positions.

## Introduction

While empirical evidence demonstrates that health and wellbeing inequalities are persistent and difficult to diminish, our understanding of the reasons for this ﻿is﻿ not clear. The study of health inequalities has largely focused on the social determinants of health such as income and education, rather than on the impact of inequal opportunities on the choice of people for a way of living they have reason to value, including their actual choices for a healthy lifestyle^1,2^. The attempts to diminish the social inequalities in health and wellbeing have been long criticized for top down and expert led approaches failing in reaching especially the most vulnerable population groups. In fact, we do not know much about their life situations^3–5^.

The capability approach, introduced by Amartya Sen and Martha Nussbaum^6–8^, has increasingly been suggested as an alternative way to look at the inequalities in health and wellbeing. The approach emphasizes the empowerment of individuals to be active agents of change both at the individual and collective level^9–11^. The evidence produced by studies using capabilities as a key concept is promising but mixed, and the mechanisms between capabilities*,* health and wellbeing remain unclear^3,12–15^. This study aims at contributing to these discussions by examining within the capability framework the determinants of quality of life (QoL) in four groups of disadvantaged people, with the expectation to further our understanding of the role of capabilities not only in these less studied population groups but also in social policy in general.

Today QoL is largely understood as a multi-dimensional concept reflecting the ways of life, where the weights of the issues of importance are changing over the life course and measured by personal profiles often summarized in overall assessments. A definition of quality of life in terms of the *experience* of individuals is most associated with the subjective well-being tradition in the behavioral sciences. The underlying assumptions are that well-being can be defined by people’s conscious experiences in the context of their own standards, that self-reported evaluations are valid as people successfully employ them in practice conducting their everyday life, and that it is possible to measure and compare these experiences^16–18^. Especially the models elaborating the associations between the quality of the societies (ensuring opportunities) and individual quality of life (integrating all dimensions) are of interest here as they address the variation of capabilities of individuals to act within the scope of inequal societal entitlements and differin*g* personal endowments^6,10,19–21^.

The capability approach to QoL argues, both normatively and empirically, that people should have the opportunities and abilities to run a life that they have reasons to value. Fair structural conditions (such as education and income) encountered in social positions and their reflection in individual conditions (such as age, gender) acquired over the life course are to ensure these opportunities and abilities^7,8,10^. The core characteristic of the capability theory is in its focus on what people are free and able to do and to be, i.e. on their abilities to convert opportunities into QoL including the capability to choose alternatives. In this sense, perceived capabilities express the confidence or hope that the perceived current QoL is open to change by one’s own agency.

Our study framework combines the theoretical frameworks of capabilities and QoL and focuses on the differential associations between sets of conditions, capabilities, and dimensions of QoL. Because actual QoL and capabilities are difficult to measure, we choose perceived capabilities and perceived QoL as the ways to address the measurement problems. Regarding perceived capabilities, we rely on the operationalizations by Anand and colleagues^12^, and with the concept of QoL, we rely on the WHO’s 4-dimensional model of physical, psychological, social, and environmental QoL^22^ as it combines both concepts of health and wellbeing. Concerning the conditions, we operationalize them as individual and structural factors impacting on individual capabilities to achieve and maintain QoL. Our focus is on four population groups which the previous studies have found as being in disadvantaged positions regarding their opportunities for achieving health and wellbeing: (1) young people outside education/employment/training (NEETs)^23,24^; (2) long-term unemployed persons^25,26^; (3) refugees at their early resettlement^27,28^; and (4) older people living alone and having wellbeing deficits^29–31^.

**Figure 1:**
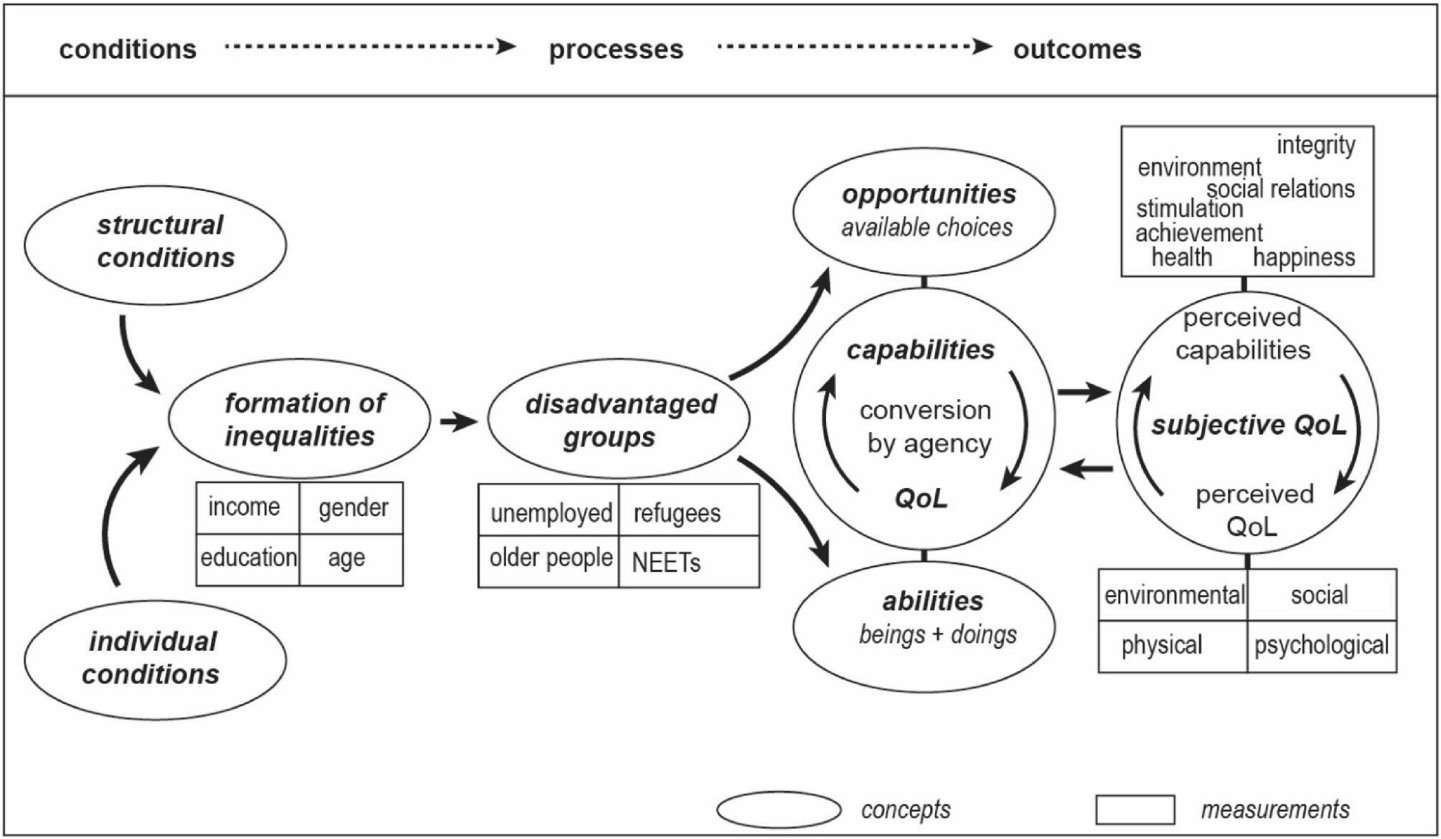
The Conceptual Model—capabilities as conversion factors between conditions and subjective QoL.

We have summarized our literature review in Fig. 1. to depict the theoretical framework of our study. The figure illustrates the idea that a person’s capabilities are strengthened or curtailed by antecedent individual and structural factors conditioning the formation of inequalities, positioning individuals in an unequal distribution of resources for health and wellbeing, and, thus, influencing the available opportunities^1,3,13,19^. This position in an inequal distribution of conditions we consider as disadvantage*,* and the four disadvantaged groups in our study may be identified by their (combination of) conditions.

Applying this theoretical framework, our research questions were to:

1. Describe the variation in the individual (age, gender) and structural (education, income) factors, perceived capabilities, and QoL in the four study groups.
2. Explore the associations of the individual and structural factors and capabilities with dimensions of QoL.
3. Examine the differences in these associations across the study groups.

## Methods

Our data and measures were taken from the existing data pool produced in the research project “Inclusive promotion of health and wellbeing” (PROMEQ, 2016–2019)^32^, and the study was performed in line with the principles of the Declaration of Helsinki*.* Approval was granted by the Research Ethics Committee of the University of Eastern Finland. All the participants received information of the study and signed informed consent. In Finland, only for participants who are below 13 years, an additional informed consent statement from legal guardians or parents is needed^33^.

**Figure 2:**
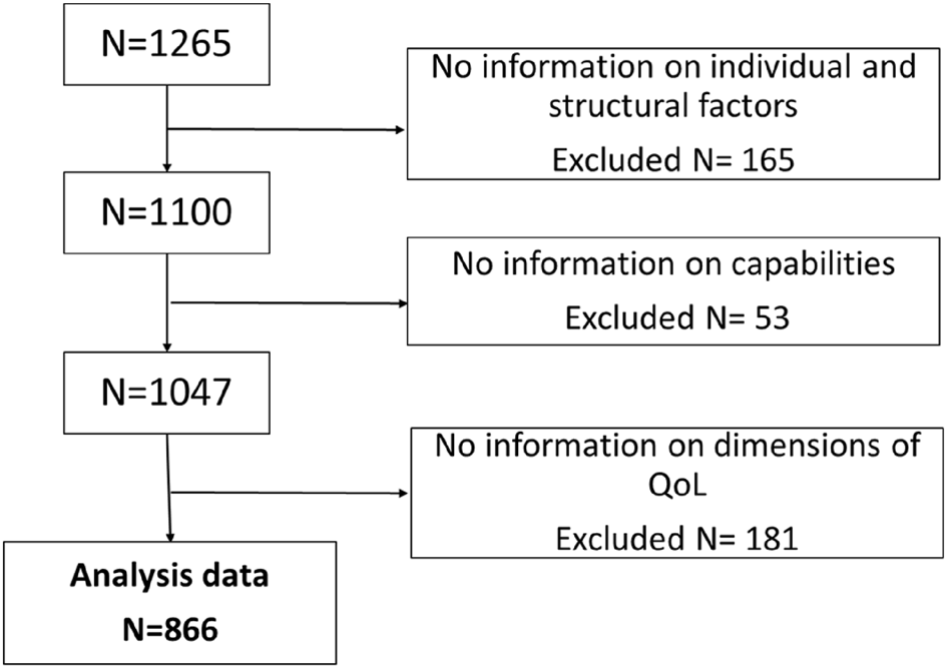
Reduction of the data.

The pooled data (N = 1265), available in the Finnish Social Science Data Archive^34^, consists of four cross-sectional and mutually exclusive subsamples, of which one is a random sample (long-term unemployed), and the rest are probability samples (NEETs, refugees, older people). Hence, the sampling allows explorative studies but not generalizations^35^. The data collection methods varied in different PROMEQ-subprojects from self-completed questionnaires to postal surveys and focus group interviews, but all used the same standardized questionnaire and research protocol. The study participants were recruited in collaboration with the social and health care professionals and NGOs relevant to each group in the three cities (Jyväskylä, Kouvola, and Vantaa) and in the two Social and Health Care Regions (Siun Sote in Southern Carelia and Eksote in Central Finland)^36^. Only those who had answered in all the studied questions (N = 866) were included in this study (Fig. 2).

The data on NEETs consists of young adults aged 16–30 years participating in the targeted youth services. The long-term unemployed persons were registered as unemployed at least for 12 months continuously and did not participate in any activation programs nor in the targeted youth services. The refugees were quota refugees or persons granted with asylum status since January 1rst, 2014, aged 18–65 years, who spoke either Arabic, Farsi-Dari, English, or Finnish, and participated in the local integration services for refugees in Eastern Finland, but not in the targeted youth or unemployment services. The data on older persons consists of full-time retired persons aged 65 years or older who lived alone with at least one subjective wellbeing deficit such as loneliness and were voluntarily enrolled in the study. The data matrix of the variables used in our analyses displays a structural variation characterizing people in different life-situations and phases of their lifecycles (Appendix 1). The values of the skewness indicate a symmetric data, and all kurtosis values except the capabilities for personal integrity are within the limits of the normal distribution^37^.

Quality of Life (QoL) was assessed using the validated Finnish language version^38^ of the internationally validated WHO QoL Bref-scale^22,39^ of self-reported QoL in four dimensions. The physical dimension covers daily pain, dependence on medication, energy, mobility, sleep, daily functioning, and workability. The psychological dimension includes evaluations of enjoying life, meaningfulness of life, concentration, bodily appearance, self-esteem, and negative feelings. The social dimension addresses satisfaction with social relations, social support, and sexual life. The environmental dimension covers feeling of safety, quality of the living environment, economic situation, access to necessary information, and satisfaction with access to health care, transportation, and leisure-time opportunities. The values of indicators are measured by the Likert scale ranging from “very poor” (= 1) to “very good” (= 5). For analyses, summary scores ranging from 0 to 100 were calculated for each dimension of quality of life according to the scoring guidelines^22^.

Capabilities were measured using the closed 8-item scale developed by Anand et al.^12,40^. The measure consists of 7 single items measuring perceived capabilities: to seek happiness; to achieve things; to live a healthy life; for intellectual stimulation; to form satisfying social relations; for being in pleasant environments (including home, work, and leisure-time); to act with personal integrity. The 8th item asks the person to evaluate his/her options taking all the previous 7 capability items into account (capabilities overall: “Taking all things together, I think my options are…”) on a scale from one (“Very Bad”) to seven (“Very Good”). The scale was translated for the purposes of the PROMEQ -study into Finnish, Arabic, Persian, and Kurdish, following the standard translation and back-translation procedures. For analyses, we used capability items coded from 0 (very bad), 1, 2, 3(neutral), 4, 5, to 6(very good).

Variables measuring individual factors included age (continuous) and gender (men = 0, women = 1). The structural factors included education (low = ” I have no studies”, “elementary school” or “comprehensive school”; middle = ”middle school”, “high school” or “vocational school or similar”; high = ”university degree”, “lower academic” or “higher academic”); and annual household income before taxes (0–10,000, 10,001–25,000, 25,001–).

Corresponding to our three research questions we used both descriptive analyses and structural equation modelling in our statistical analyses, and conducted them with STATA 15.1^41^. The descriptive analyses (means, standard deviations, Pearson Chi2 tests for group differences) were used to describe the variation of individual and structural factors, capabilities and QoL by dimensions in our study population. Correlation analyses were employed to preliminarily examine the connections between individual and structural factors, capabilities and QoL dimensions, and the multicollinearity of the examined variables^42^. The correlation matrix (Appendix 2) shows that correlations between the QoL dimensions were between 0.2 and 0.6, and within the 8 capability-items between 0.3 and 0.6, so too high multicollinearities (> 0.7) were not observed, as demonstrated also in the regression collinearity test.

To track the direct and indirect effects we employed structural equation modelling (SEM) with capabilities as the theoretical construct of a latent variable^43^. We used the maximum likelihood value to estimate standardized regression coefficients and produced robust standard errors. First, we estimated the measurement model (a latent variable) for capabilities using only the seven single capabilities of the scale, and calculated the model fit index (standardized root mean squared residuals = SRMR), where the cut-off criteria lower or close to 0.08 demonstrates a good model fit^44^. We also calculated Cronbach’s alpha (≥ 0.8 indicates good level of reliability) for the measurement model to examine internal consistency of the latent variable. Second, we estimated the structural model to examine the direct, indirect, and total effects (= sum of direct and indirect effects) of individual and structural conditions and capabilities (latent variable) on the four dimensions of QoL. Finally, we examined the group-differences in these effects by using the group status (NEET, long-term unemployed, refugees, older people) as the grouping variable.

## Results

Our data and measures were drawn from a previous Finnish study on four mutually exclusive groups characterized as disadvantaged in their social positions^32,34^. Addressing our first research question, we analyzed our data (N = 866) with descriptive statistical methods (Table 1).

**Table 1:** Descriptive results of the examined groups, PROMEQ baseline data 2017, N = 866.

	(1) NEETS			(2) Long-term unemployed			(3) Refugees			(4) Older people			
Individual factors	N	M	SD	N	M	SD	N	M	SD	N	M	SD	p-value
Age in years	84	24	3.10	433	53	10.19	90	34	9.49	259	77	7.53	
Gender	N	%		N	%		N	%		N	%		***
Men	36	43		230	53		56	62		52	20		
Women	48	57		203	47		34	38		207	80		

**Structural factors**	N	%		N	%		N	%		N	%		***
Education													***
Low	30	36		88	20		26	29		79	31		
Middle	30	36		251	58		23	26		117	45		
High	24	39		94	22		41	46		63	24		
Household income													***
0–10,000	46	55		188	43		41	46		19	7		
10,001–25,000	27	32		136	31		44	49		159	61		
25,001-	11	13		109	25		5	5		81	31		

Capabilities for	N	M	SD	N	M	SD	N	M	SD	N	M	SD	
Happiness	84	3.92	2.00	433	2.95	2.14	90	2.49	2.65	259	2.63	2.22	
Sense of achievement	84	3.61	1.92	433	2.79	2.03	90	2.94	2.53	259	2.54	2.11	
Health	84	3.76	2.03	433	3.39	2.12	90	3.08	2.69	259	3.42	2.09	
Intellectual stimulation	84	3.87	2.03	433	3.52	2.17	90	2.89	2.60	259	3.64	2.04	
Social relations	84	3.83	1.81	433	3.29	2.04	90	2.71	2.50	259	2.99	2.10	
Environment	84	3.98	1.94	433	3.46	2.12	90	2.90	2.57	259	3.81	2.01	
Personal integrity	84	4.57	1.62	433	4.40	1.93	90	4.11	2.62	259	4.82	1.73	
Capabilities overall	84	3.69	2.11	433	3.50	2.10	90	3.67	2.56	259	3.57	2.01	

Quality of Life (QoL, Scale: 0–100)	N	M	SD	N	M	SD	N	M	SD	N	M	SD	
Physical QoL	84	66.1	17.3	433	62.8	20.3	90	70.2	18.8	259	58.4	18.0	
Psychological QoL	84	54.1	19.2	433	57.7	20.2	90	65.3	18.9	259	59.6	17.2	
Social QoL	84	65.3	20.2	433	60.0	23.2	90	68.2	21.6	259	60.6	19.4	
Environmental QoL	84	63.6	14.1	433	60.5	17.6	90	62.6	16.3	259	64.9	15.8	

Number of participants (N), Mean (M), standard deviation (SD), percentage (%) and Pearson Chi2 statistical significance of group differences (p-value).

NEETs were young adults with the average age of 24 years (range 17–31) participating in the targeted youth services (outreach youth work, one-stop guidance centers, youth workshops). The long-term unemployed persons were in average 53 years old (range 22–66) and registered as unemployed at least for 12 months continuously and did not participate in any activation programs. The refugees*,* with the average age of 34 years (range 19–61) were quota refugees or persons granted with asylum status since January 1rst, 2014, who spoke either Arabic, Farsi-Dari, English, or Finnish, and participated in the local integration services for refugees in Eastern Finland. The older persons with the average age of 77 years (range 61–100) were full-time retired and lived alone with at least one subjective wellbeing deficit such as loneliness and were voluntarily enrolled in the study. Men and women were almost equally presented in all groups except in older persons, among whom 80% were women. The NEETs reported most often lowest level education, middle level was most common for unemployed and older people, and highest level for refugees. The majority had yearly household income less than 25,000€, and a level higher than this was rare and most common for older people and unemployed. Group differences were statistically significant.

Regarding capabilities*,* the *NEETs* scored best in all items except their scope to act with personal integrity and their overall capabilities scored second best. Long-term unemployed scored lowest in their sense of achievement, quite low in their capabilities for healthy life, and their overall capabilities scored low. The refugees evaluated almost all their capabilities lower than other groups, but their overall capabilities as best. Older people had lowest scores in happiness and sense of achievement, and their social relations and overall capabilities scored also low. However, they perceived their capabilities for living in pleasant environments and to act with personal integrity high. In general, variation in the perceived capabilities was quite small in terms that all their evaluations focused on the lower end of the scale (0–8), except of personal integrity with mean values over 4.

Looking at the dimensions of QoL*,* on average, refugees scored highest in all dimensions except the environmental one. In physical dimension, older people scored lowest, and NEETs second lowest. Psychological dimension scored lowest in NEETs, but the values were low also in long-term unemployed and older people. Social QoL scored lowest in the long-term unemployed and older people. In the environmental dimension, NEETs scored best and older people lowest. There was more variation in physical and environmental QoL than in other dimensions among the groups, and refugees showed most in-group variation.

**Figure 3:**
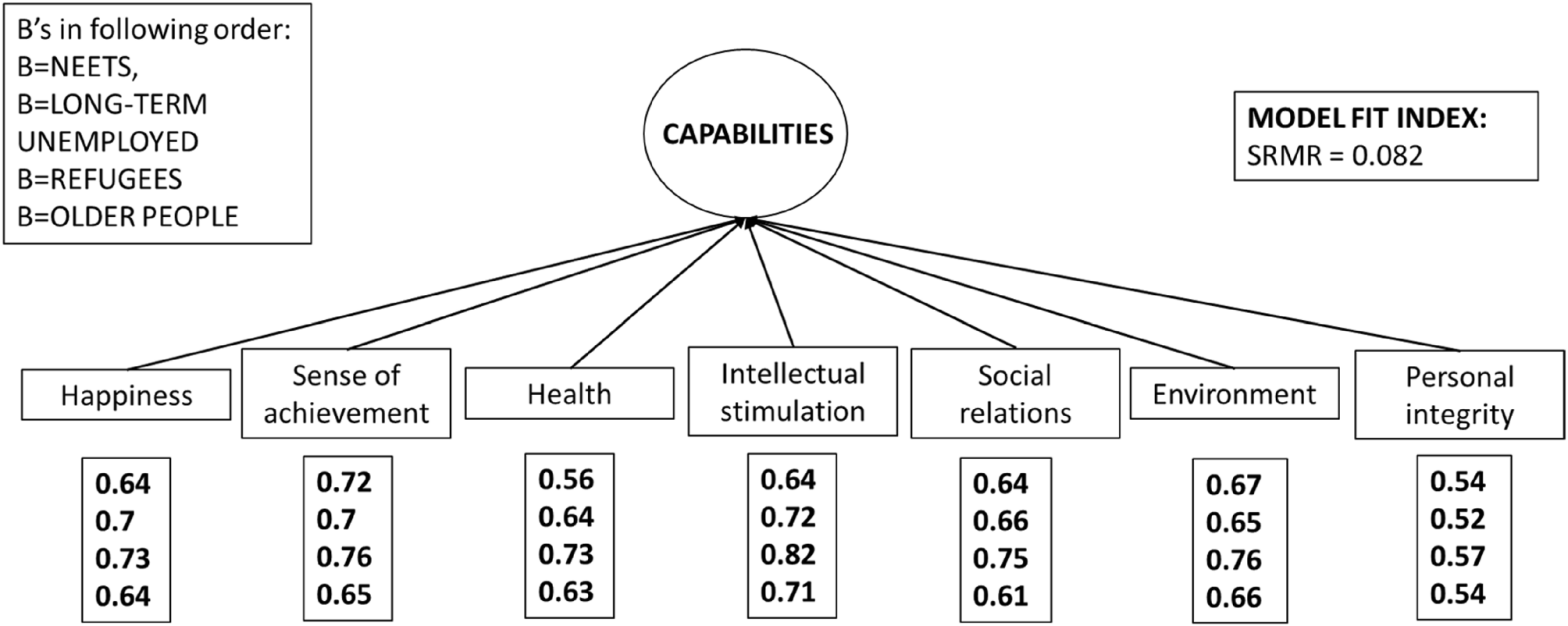
Measurement model for capabilities (latent variable) in the four study groups, standardized item loadings for the latent variable, and model fit index (SRMR), PROMEQ data (N = 866).

To analyze the relations between the different capabilities and dimensions of QoL we constructed first the measurement model of capabilities (Fig. 3). The model demonstrated a good latent structure with seven individual items of capabilities. The model fit SRMR = 0.082 (threshold = 0.8) indicated a good model fit, and the Cronbach’s alpha value 0.84 (threshold = 0.8) a good internal consistency. The coefficient values showed each single capability scoring high in all the studied groups, and especially in the refugees, indicating the importance of each capability for QoL in these groups.

The measurement model of capabilities (Fig. 3) demonstrated a good latent structure with seven individual items of capabilities for disadvantaged groups. The model fit SRMR = 0.082 indicates a good model fit, and the Cronbach’s alpha value 0.84 a good internal consistency. Based on the values of the coefficients of the measurement model we can see that each single capability get high values of coefficients in all the studied groups, the group of refugees having the highest values in each capability. Looking by a group, the two most important capabilities among NEETs were sense of achievement and ability to live in pleasant environments, but social relations, intellectual stimulation and happiness gained also high values. The capabilities for intellectual stimulation, happiness, and sense of achievement were the three most important for the long-term unemployed people. For refugees, almost all capabilities scored high, the three key capabilities being intellectual stimulation, sense of achievement, and pleasant environment. Older people tended to have lower values in almost all capabilities, and the three most important for them were intellectual stimulation, pleasant environment, and sense of achievement.

**Figure 4:**
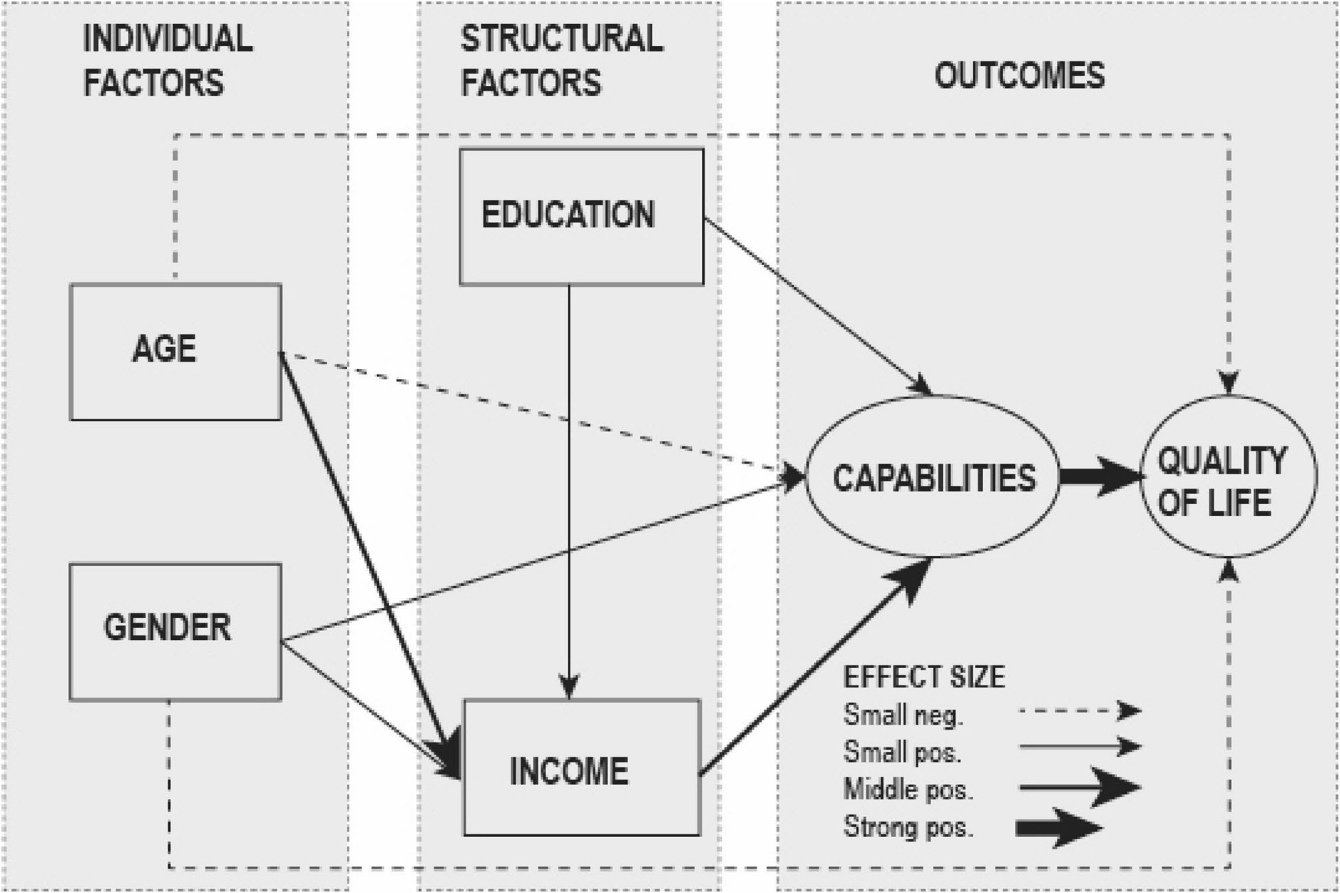
A simplified presentation of the effects of individual and structural factors and capabilities on different dimensions of QoL in the examined groups. SEM-modelling with latent variables, PROMEQ data (N = 866).

Next, we used SEM-modelling to track the direct, indirect, and total effects of the individual and structural factors and the latent construction of capabilities on the four dimensions of QoL in our study population in general. The statistically significant effects (p < 0.05) and effect sizes are demonstrated using different types of arrows (Fig. 4). The model fit was good for all dimensions of QoL and explained 44–57% of the variation in the dimensions of QoL in our data.

Figure 4 illustrates the strong positive and direct effect of perceived capabilities on QoL in our study population. The figure also illustrates how the examined individual and structural factors, especially age and income shaped the individual capabilities for QoL. Age had a small negative direct effect (standardized regression coefficient B = − 0.1) on capabilities whereas the direct effect of female gender was small but positive (B =  + 0.1). Age had a middle size positive direct impact (B = 0.3) on household income whereas the impact of female gender was smaller (B = 0.1). Household income in their turn had a middle size direct positive effect (B = 0.3) on capabilities for QoL. As expected, education had direct small positive effect (B = 0.2) both on income and capabilities (B = 0.1). The model fit (SRMR) ranging between 0.4–0.5 was good for all dimensions of QoL, and the models explained 44–57% of the variation in the dimensions of QoL in our data.

Table 2 shows in more detail the strong direct effects of capabilities on different QoL dimensions and illustrates the much smaller impacts of individual and structural factors. The B-values of capabilities indicated the examined capabilities being statistically slightly more significant for psychological and environmental QoL.

**Table 2:** The direct, indirect, and total effects of the examined capabilities and individual and structural factors on different QoL dimensions, standardized regression coefficients (B), only statistically significant shown (p < 0.05), PROMEQ data (N = 866).

	Direct effects				Indirect effects				Total effects			
	Physical QoL	Psycho-logical QoL	Social QoL	Environ-mental QoL	Physical QoL	Psycho-logical QoL	Social QoL	Environ-mental QoL	Physical QoL	Psycho-logical QoL	Social QoL	Environ-mental QoL
Capabilities	0.5	0.6	0.5	0.6								
**Individual factors**												
Age	− 0.1	0.1	− 0.1	0.1	− 0.1	− 0.1	− 0.1	− 0.1	− 0.2		− 0.1	0.1
Gender (female)	− 0.1	− 0.1			0.1	0.1	0.1	0.1				
**Structural factors**									0.3	0.3	0.4	0.4
Education			− 0.1		0.1	0.1	0.1	0.1				
Household income			0.1	0.1	0.2	0.2	0.2	0.2				

From the individual factors, age had a direct negative effect on physical and social QoL whereas the effect was positive on psychological and environmental QoL. Female gender affected negatively physical and psychological QoL. Indirectly age had a negative effect on all QoL dimensions whereas female gender had a direct positive effect. From the structural factors, the household income impacted directly on QoL more than education, and income were important especially for social and environmental QoL. The total effects of structural factors on QoL were higher than those of the individual factors, reflecting the disadvantages in education and income in our study population.

Regarding the direct effects on the physical QoL*,* the capabilities showed a strong positive (B = 0.5) effect, whereas the effects of age and gender were small and negative. The direct effects of education and household income were not statistically significant (Fig. 4). Regarding the indirect effects*,* age had a small negative effect, gender a small positive effect, and household income and education both had small positive indirect effects through capabilities. The estimated total effect of personal conditions was small and negative, whereas the total effect of structural conditions was middle size and positive.

Regarding the direct effects on psychological QoL, capabilities had a strong positive direct, whereas the effect of age was small and positive, and the effect of gender small and negative. Education and income were not statistically significant. The indirect effect of age was small and negative, whereas gender, education and household income all had small positive indirect effects through capabilities. Total effect was not found for personal conditions, whereas social conditions had a medium size positive effect on psychological QoL.

Regarding the social QoL, the direct effect of capabilities was strong and positive. The direct effects of age and education were small and negative, whereas household income was a small but positive direct effect. The effect of gender was not statistically significant. The indirect effect of age was small and negative, but gender, household income and education all had small positive indirect effects through capabilities. The total effect of personal conditions was small negative whereas the total effect of structural conditions was medium positive.

For environmental QoL, the direct effect of capabilities was strong, and the effects of both age and household income were small but positive. The effects of gender and education were not statistically significant. Regarding the indirect effects*,* age had a small negative effect whereas gender, education, and household income all had small positive indirect effects through capabilities. The total effect of personal conditions was small positive the total effect of social conditions was medium positive.

Finally, we employed the SEM-modelling to analyze the group-differences in our study population in the direct and indirect associations of individual and structural factors and capabilities with the four dimensions of QoL (Table 3). The SEM-model demonstrated reasonably good fit to the data in all the models (0.08–0.09). Again, we report the direct, indirect, and total effects by each dimension of QoL. Only significant effects between the examined variables are included in the estimations.

**Table 3:** Group differences in the associations of individual and structural factors as well as capabilities with dimensions of QoL, explanation rates (R2) and model fit index (SRMR), statistically significant (p < 0.05) coefficients in bold, PROMEQ data (N = 866).

	Physical QOL				Psychological QOL				Social QOL				Environmental QOL			
	NEETS	Long-term unemployed	Refugees	Older people	NEETS	Long-term unemployed	Refugees	Older people	NEETS	Long-term unemployed	Refugees	Older people	NEETS	Long-term unemployed	Refugees	Older people
**Direct effects**																
Capabilities	**0.5**	**0.5**	**0.6**	**0.5**	**0.6**	**0.6**	**0.7**	**0.7**	**0.5**	**0.5**	**0.6**	**0.5**	**0.5**	**0.6**	**0.6**	**0.6**
Age	0.1	0.01	**− 0.3**	0.03	− 0.03	0.1	− 0.005	**0.2**	**0.2**	0.1	0.2	0.01	− 0.02	0.02	− 0.03	**0.2**
Gender (female)	**− 0.3**	− 0.1	− 0.1	− 0.1	− 0.1	0.02	**0.2**	**− 0.1**	0.03	0.04	0.03	**0.2**	− 0.02	− 0.1	0.03	− 0.01
Education	0.1	0.1	**0.2**	− 0.04	0.04	**− 0.1**	**0.2**	− 0.03	− 0.2	**− 0.2**	0.1	**− 0.2**	0.1	− 0.1	0.1	− 0.1
Household income	0.03	− 0.1	0.2	− 0.001	− 0.1	0.02	**0.02**	**− 0.1**	− 0.2	− 0.1	− 0.1	**0.03**	0.1	**0.1**	0.1	0.1
Indirect effects*																
Age- > capabilities	0	**− 0.05**	0	**− 0.05**	0	**− 0.06**	0	**− 0.07**	0	**− 0.05**	0	**− 0.05**	0	**− 0.06**	0	**− 0.06**
Gender- > capabilities	0	0	0	0	0	0	**0**	**0**	0	0	0	0	0	0	0	0
Education- > capabilities	**0.15**	0	0	**0.1**	**0.18**	**0**	0	**0.14**	**0.15**	0	0	**0.1**	**0.15**	0	0	**0.12**
Household income- > capabilities	**0.15**	**0.15**	0	**0.1**	**0.18**	**0.18**	0	**0.14**	**0.1**	**0.15**	0	**0.1**	**0.15**	**0.06**	0	**0.12**
Total effects of*																
Individual factors (age + gender)	**− 0.2**	**− 0.05**	**− 0.3**	**0.45**	0	**− 0.06**	**0.2**	**0.03**	**0.2**	**− 0.05**	0	**0.15**	0	**− 0.06**	0	**0.14**
Structural factors (education + household income)	**0.3**	**0.15**	**0.2**	**0.2**	**0.36**	**0.08**	**0.22**	**0.18**	**0.25**	**− 0.05**	0	**0.03**	**0.3**	**0.16**	0	**0.24**
Explanation rate (R^2^)	60%	39%	70%	44%	60%	64%	80%	66%	59%	47%	60%	55%	62%	55%	68%	60%
Model fit (SRMR)	0.08				0.09				0.08				0.09			

Total effect = direct effects + indirect effects.

*Only significant effects between the examined variables were included into calculation of the indirect and total effects.

The group-based SEM-modelling confirms the previous finding on the examined capabilities having strong positive direct effects on all dimensions of QoL in each study group, and only minor group-differences were observed. However, the effects of the examined individual and structural factors varied more. Age associated negatively with physical QoL in the refugees, but positively with social QoL in the NEETs, and with psychological and environmental QoL in older persons. Female gender had a negative relation with physical QoL in the NEETs and in older persons, but positive with psychological QoL in the refugees and with social QoL in older persons. Education associated positively with physical and psychological QoL in the refugees, but negatively with psychological and social QoL in the long-term unemployed, and with social QoL in older people. Household income had positive direct effects on psychological QoL of refugees, on social QoL of older people, and on environmental QoL in the long term employed, but the effect was negative on psychological QoL in older people.

Some variation in the indirect effects were also observed between the study groups (Table 3). Age had indirect effect through capabilities on all dimensions of QoL in the long-term unemployed and in older persons. Education impacted through capabilities in all QoL dimensions in the NEETs and in older people, and household income impacted on all QoL dimensions in the NEETs, long-term unemployed and older people. Regarding the estimated total effects, the impacts of age and gender (individual factors) were strongest on older people’s physical, social, and environmental QoL. The total effects of education and income (structural factors) were observed in all QoL dimensions in all groups, and especially in the NEET’s. In comparison with other study groups, the effects of these structural factors were strong also on psychological QoL in the refugees, and on environmental QoL in older people.

## Discussion

The health and wellbeing of disadvantaged groups have rarely been examined utilizing a holistic and multidimensional approach distinguishing and combining individual and structural factors, capabilities, and QoL (Fig. 1). The results describe the structural disadvantages, lowered QoL, and limited perceived capabilities of four disadvantaged groups, i.e. NEETs, long-term unemployed, refugees, and older persons living alone with wellbeing deficits. In our study population, low income was typical, but the education level varied, low education being most typical for the NEETs and higher level for the refugees. Our study participants evaluated their QoL in all four dimension lower than in the Finns in average^45^, and their perceived capabilities also were located on the lower end of the scale we used. Within groups the factor age was not significant, since the groups were rather homogeneous and stratified over the life course. The differences within groups in gender, education, and income were statistically significant but the differences in perceived capabilities and QoL were not.

Previous studies have raised questions about the mediating role of capabilities, on how capabilities should be measured, and whether it is important to possess some basic capabilities, or is there some certain threshold of capabilities^14^. Our results are novel in that they show the examined capabilities to affect strongly, positively, and directly all QoL dimensions in all the examined groups, supporting the idea that the possession of some basic capabilities and the belief in one’s own agency having choices are important factors shaping individual QoL.

In this perspective, it is also to be expected that the individual factors (age, gender) which are ascribed by society and not seen as individual achievements show a weaker effect on capabilities and QoL than the structural factors (income, education) which are influenced by perceived opportunities and personal choices. For all, most important capabilities were the abilities to achieve things in life (sense of achievement) and to get intellectual stimulation. Being able to live in pleasant environments (taking home, work, and leisure-time into account) was important for the NEETs, refugees, and older people; the ability to build up social relations for the NEETs; and all but older people perceived happiness as important for their QoL. Our results emphasize the important mediating role of capabilities in achieving a good life and reveal some similarities and differences across the groups. However, we could not identify any certain threshold of capabilities necessary for good QoL, perhaps because the perceived capabilities of our study participants tended to be on the lower end of the scale, and they evaluated their QoL lower than the Finnish population in general^45^.

Considering the direct effects of the individual and structural factors by groups, age and gender impacted on at least one dimension of QoL in all groups except the long-term unemployed. In the NEETs*,* the positive association of age with their social QoL indicated that the older a NEET was (range 16–30 years) the better were his/her social relations. Female gender´s negative association with physical QoL suggests older female NEETs having poorer physical health than the younger ones. In the refugees, the negative association of age with physical QoL suggests older refugees to have worsened physical health, but female gender contributed positively to psychological QoL in this group. In older people, age associated directly positively with psychological and environmental QoL, and female gender positively with social QoL, indicating perhaps their beneficial adaptation with old age and especially older women having better social wellbeing^29^. Education associated directly and positively on at least one QoL dimension in all except the NEETs with whom the lowest education level was most common. The association was negative with psychological and social QoL in the long-term unemployed, suggesting that the better educated of them may be more vulnerable for problems with mental health and social relations. In the refugees, education associated positively with physical and psychological QoL, and better income with psychological QoL, indicating the importance of these structural factors for QoL in this group. In older people, education and income impacted positively on social QoL but negatively on psychological, suggesting the structural factors supporting social wellbeing in this group, but more educated older people being vulnerable for lowered psychological wellbeing. Previous studies have found, for example, meaningful life to be one of the important determinants of QoL in later life^46^.

Considering indirect effects of individual and structural factors, gender did not have statistically significant effects, but age impacted negatively through capabilities in the long-term unemployed and older people, suggesting their perceived capabilities for all QoL dimensions to decrease with age. Education associated positively with all dimensions of QoL in the NEETs and older people, suggesting that better education contributed to their capabilities for QoL. Income impacted through capabilities positively with all QoL dimensions in all groups but refugees. In the long-term unemployed, better income was in positive relation with environmental QoL, which is to be expected as this QoL dimension included a question on economic situation of the respondent. The indirect effects of the examined structural factors through capabilities were strongest in the NEETs and older people, indicating the importance of these structural factors for their capabilities for a good life.

The total effect of structural factors (education, household income) was stronger than that of individual factors (age, gender) in all study groups, showing the importance of social position for perceived QoL. The structural factors played an important role for several dimension of QoL especially in the NEETs. In older people, the positive total effect of individual factors was highest, and focused on their physical and environmental QoL, and both structural factors associated positively with all QoL dimensions in this group. The result is partially at odds with previous findings on that older ages and female gender decrease especially physical QoL^20,29,46^. Our result may indicate that better education and income contribute to better QoL and to the possession of the necessary capabilities, and these together may help older people to adapt to their lowered physical health and functioning. Across the entire study population, the total effects of the structural factors were in general stronger than that of the individual factors.

The group variation in the weights of different factors impacting QoL in our study population gives support to our initial assumption that individual and structural factors may both strengthen and limit the development and use of person’s own capabilities to achieve a good life^3,12,13,15^. It is a well-established result that individual and structural factors have a profound impact on many areas of health and wellbeing^2,10,16^ and that disadvantage is negatively associated with QoL^26,47^. Previous research have found the NEETs being from multiple perspectives vulnerable for several health and wellbeing risks, such as low social support^48^, poor financial situation^49^, youth unemployment^50^, and risk of social withdrawal^51^. Low QoL among long-term unemployed may be linked with problems of stigmatization and self-approval^25,26^, and the problems the refugees face might be related to their previous hazardous situations^27,28^, or to the initial problems in finding out their ways of living in new societies^52^. The previous studies have also found that higher education and better income provide older people with better opportunities for active leisure-time, and for forming satisfying social relations that are relevant for psychological and social wellbeing^29,53,54^. It is also well established in previous literature that especially older women with small income and living alone are vulnerable for different wellbeing deficits such as lack of recognition, loneliness, and weak social networks, multimorbidity, poverty and unmet care needs, and that they may benefit of support and encouragement for vitalizing their QoL^29,38,46,53,54^.

Although our data and methods do not allow for causal generalizations, our models of the determinants of QoL within the capability framework confirm the important mediating role of capabilities, and direct attention to the importance of structural factors for health and wellbeing. Following the capability approach^3,28,35^, our results suggest that to reduce inequalities in health and to promote wellbeing, policies should focus on improving both the opportunity structures—as far as they are subject to social policies programs—and the capabilities of people in disadvantaged positions to cope with the challenges posed by changing structural (education, income) and individual (age, gender) factors over the life course. These together would strengthen their agency for QoL converting opportunities and abilities into improvements and adjustments.

The capability framework calls for quite a different strategy for promotion of health and wellbeing as is currently in use in Finland. The traditional top-down health education fails to offer realistic and tailored options for disadvantaged people who may vary greatly in their possessions of capabilities^4,36^. While there is a need for general support of disadvantaged people, there is also a need to respond to the diversity of capabilities in taking advantage of this support. The capability framework calls for respect for persons with lowered capabilities for QoL, and for activities that correspond to their differing needs. A prerequisite for meeting these conditions is a responsive bottom-up strategy working with an integrated framework of health and welfare policies. On first sight, this way may appear to be consuming more time and other resources, but it will prove to be more effective for the individuals and for the society in general^32,36,55^.

Regarding strengths and limitations, this study is one of the first empirical explorations of the capability approach to QoL in Finland. The PROMEQ cross-sectional data^32^ provided us with a unique opportunity to examine the associations between capabilities and QoL among disadvantaged population groups. For QoL, we had national reference values available for comparisons^45,55^ but similar analyses were not possible for capabilities as the reference values are lacking. The PROMEQ-study aimed at ensuring the validity and reliability of the study by using standardized measures and study protocol^32^, but still, the respondents may have interpreted the meanings of the survey instruments differently regardless of the use of trained co-researchers in the interviews. Further, the focus on disadvantaged groups limited the opportunities for random sampling as any register or list of people in “disadvantaged positions” is not existing. Although a cross-sectional study with purposive sampling cannot establish causality or allow straightforward generalization of results, our pooling design, supported by statistical testing allows the exploratory investigations we have performed in this study. Our results provide indicative evidence for both direct and mediating role of capabilities to QoL, which should be further examined in future studies.

Further research with larger samples with validated measures and preferably longer follow-up studies with repeated measurements are necessary for establishing the causal mechanisms between capabilities and dimensions of QoL. It would be also valuable to examine other disadvantaged groups such as disabled people and include health as an independent individual factor as it was now included as part of the measure of QoL. Also, exploring with other capability measures should tackle the difficult conceptual and measurement problem whether perceived capabilities correspond to the available opportunities on the individual level.

## Supplementary Information

Supplementary Information.

## Data Availability

The PROMEQ data is freely available from the Finnish Social Science Data Archive (FSD) for research, teaching and study purposes: https://services.fsd.tuni.fi/catalogue/FSD3436. To obtain the data, you must register for the FSD and supply purpose of the data use. For support, you can contact FSD User Services: user-services.fsd@tuni.fi.
