# Supplementary material for: Exploring the capability approach to quality of life in disadvantaged population groups
Source: Sci Rep. 2022 Sep 15;12:15248. doi: 10.1038/s41598-022-18877-3 (PMC9477846; doi:10.1038/s41598-022-18877-3)
Supplement: Supplementary file 1 — Supplementary Information. [file 41598_2022_18877_MOESM1_ESM.docx]

**SUPLEMENTARY MATERIALS**

**APPENDICES:**

*APPENDIX 1. Description of data and variables used in the analyses, PROMEQ data 2017, N=866*

*APPENDIX 2 . Correlation matrix between the examined variables**
